# Obscenity Detection Using Haar-Like Features and Gentle Adaboost Classifier

**DOI:** 10.1155/2014/753860

**Published:** 2014-06-05

**Authors:** Rashed Mustafa, Yang Min, Dingju Zhu

**Affiliations:** ^1^Shenzhen Institutes of Advanced Technology, Chinese Academy of Sciences, Shenzhen 518055, China; ^2^University of Chinese Academy of Sciences, Beijing 100049, China; ^3^Department of Computer Science and Engineering, University of Chittagong, Chittagong 4331, Bangladesh; ^4^Department of Computer Science, The University of Hong Kong, Hong Kong 999077, Hong Kong; ^5^School of Computer Science, South China Normal University, Guangzhou 510631, China

## Abstract

Large exposure of skin area of an image is considered obscene. This only fact may lead to many false images having skin-like objects and may not detect those images which have partially exposed skin area but have exposed erotogenic human body parts. This paper presents a novel method for detecting nipples from pornographic image contents. Nipple is considered as an erotogenic organ to identify pornographic contents from images. In this research Gentle Adaboost (GAB) haar-cascade classifier and haar-like features used for ensuring detection accuracy. Skin filter prior to detection made the system more robust. The experiment showed that, considering accuracy, haar-cascade classifier performs well, but in order to satisfy detection time, train-cascade classifier is suitable. To validate the results, we used 1198 positive samples containing nipple objects and 1995 negative images. The detection rates for haar-cascade and train-cascade classifiers are 0.9875 and 0.8429, respectively. The detection time for haar-cascade is 0.162 seconds and is 0.127 seconds for train-cascade classifier.

## 1. Introduction

Online video and images are now easily accessible due to availability of high-speed Internet and rapid growth of multimedia technology. A report shows that a large number of teens and children search pornographic contents everyday [1]. This is a threat for the society and a concern of Internet safety. Taking care of this issue, scientists are working hard and initiated different filter techniques to screen malicious contents. Most techniques were texts-based and could not identify objectionable materials from the sites appropriately. The reason for this is that there are countless websites which do not contain sensitive texts; hence, content-based image processing especially identifying obscenity has now been a challenging research area. It has been almost two decades when Forsyth et al. [2] published the first paper in this issue on “Finding Naked People.” After that, a large number of works were accomplished by different researchers all around the globe [2–4]. The prior works concentrated mainly on skin color, which is not suitable because of skin-like objects and partially exposed images that are not considered obscene.

In this paper we focused on nipple detection for identifying objectionable images from pornographic sites. It is a challenging task because nipples are nonrigid objects varying in shape, size, scale, illumination, and partial occlusion [5]. The appearance also differs due to different ethnicity. Considering the above factors, in this research we extracted haar-like features from some cropped nipple images and used Gentle Adaboost (GAB) haar-cascade classifier for ensuring accuracy; in addition we have compared it with train-cascade classifier in order to satisfy detection time. It has been shown that haar-cascade classifier is suitable for accurately detecting nipples, but for ensuring faster detection and little accuracy train-cascade classifier is better.

The rest of this paper can be organized according to the following ways: in Section 2 some related work will be discussed, some background knowledge including color model, haar-like features, and Gentle Adaboost algorithm has been illustrated in Section 3, experimental setup will be elucidated in Section 4, results will be analyzed in Section 5, and finally a discussion in Section 6 concludes the paper.

## 2. Literature Review

Content-based image processing for identifying objectionable materials is not a new idea. The first paper was published more than twenty years ago [2]. In the past, research on this ground was followed using skin color model. A large percentage of skin was used as a measure of pornographic contents [2–9]. But due to large varieties of skin-like objects this only technique is not suitable.

There is a suitable idea to find objectionable material which is nipple detection. Nipples are considered erotogenic human body parts and have unique characteristics in all pornographic images. Fuangkhon et al. [5, 10–12] presented an object detection using image processing and neural network entitled “*nipple detection for obscene pictures*.” The authors claimed that the detection rate was 65.4%; so far it was the only paper on nipple detection until 2010. In 2010 Wang et al. [9] proposed another robust method entitled “*Automatic Nipple Detection Using Shape and Statistical Skin Color Information*;” in this paper a new approach on nipple detection for adult content recognition has been presented and it combines the advantages of Adaboost algorithm, that is, the rapid speed in object detection and the robustness of nipple features for adaptive nipple detection. The detection rate of this approach was 75.6%. Kejun et al. [7] proposed another method called “*Automatic Nipple Detection Using Cascaded AdaBoost Classifier*.” In this research they used extended haar-like features, color features, and texture and shape features to train and obtain cascaded Adaboost classifier. The authors claimed that the detection rate was 90.37%. There are some other methods of nipple detection, but this is limited for digital mammogram. According to the literature, those above-mentioned three works were significant for nipple detection research, which was devoted to identify objectionable materials from images. All works have lacked appropriate quantitative measures to classify whether an image contains nipple objects or not.

## 3. Background Knowledge

In this section significant skin color model, haar-like features, and Gentle Adaboost algorithm will be discussed.

### 3.1. Color Model (YCbCr)

In this research we used YCbCr color model for skin filtering. It belongs to orthogonal color spaces, which reduce the redundancy present in RGB, and color channels and it represents the color with statistically independent components [6]. The components are luminance and chrominance that are explicitly separated and lead to the suitability of skin color detection. YCbCr can be obtained from RGB color transformation. The color space transformation is assumed to decrease the overlap between skin and nonskin pixels, which in turn makes the process robust thereby aiding skin-pixel classification under a wide range of illumination conditions. YCbCr is an encoded nonlinear RGB, commonly used by European televisions and for image compression. Here, the color is represented by luma (which is luminance or brightness) computed from nonlinear RGB constructed as a weighted sum of the RGB values and two color difference values Cb and Cr that are formed by subtracting the luma value from red and blue components of RGB model. The following equations are the transformation from RGB to YcbCr [2–5]:

(1) $$\begin{array}{c} \text{Y}=0.299\text{R}+0.587\text{G}+0.114\text{B},\\ \;\text{Cb}\;=\text{R}-\text{Y},\\ \;\text{Cr}\;=\text{B}-\text{Y},\\ \left[\begin{array}{ccc} \text{Y} & \text{Cb} & \text{Cr} \end{array}\right]=\left[\begin{array}{ccc} \text{R} & \text{G} & \text{B} \end{array}\right]\left[\begin{array}{ccc} 0.299 & -0.168935 & 0.499813\\ 0.587 & -0.331665 & -0.418531\\ 0.114 & 0.50059 & -0.081282 \end{array}\right]. \end{array}$$

This model is suitable for use under some predefined conditions within specific systems. The Y component describes brightness and the other two values describe a color difference rather than a color itself, making the color space unintuitive. The transformation simplicity and explicit separation of luminance and chrominance components make this color space perfect for skin color modeling. In YCbCr the RGB components are separated into luminance (Y), chrominance blue (Cb), and chrominance red (Cr). And thus YCbCr space is one of the most popular selections for skin detection and has been used by many researchers [6, 8, 13].

### 3.2. Haar-Like Features

**Figure 1:**
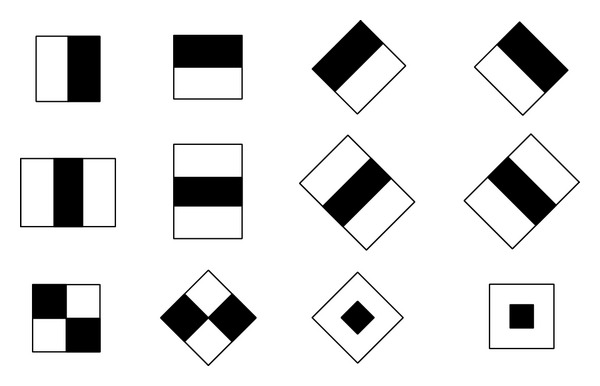
Simple haar-like features.

Haar-like features are applicable to classify generic objects. They are particularly familiar for face detection, where the system determines whether an object is a generic face. Simply knowing that an object is a face is useful for segmenting the image, narrowing down a region of interest, or simply doing some other fun tricks [14, 15]. Technically, haar-like features refer to a way of slicing and dicing an image to identify the key patterns. The template information is stored in a file known as a haar-cascade, usually formatted as an XML file [14]. This requires a fair amount of work to train a classifier system and generate the cascade file. Some simple haar-like features are described in Figure 1.

The calculation method of haar-like features is faster by introducing integral image or summed area table [16]. This is the reason that haar-cascade and train-cascade classifiers are computing features very quickly.

#### 3.2.1. Integral Image

**Figure 2:**
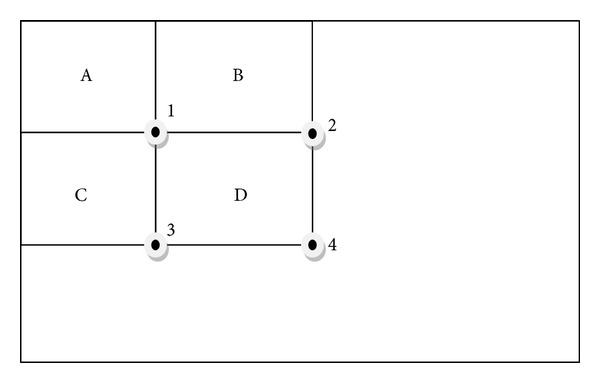
Calculation of summed area table.

Rectangular two-dimensional image features can be computed rapidly using an intermediate representation called the integral image [17]. The integral image, denoted by *ii*(*x*, *y*), at location (*x*, *y*) (2)-(3) contains the sum of the pixel values above and to the left of (*x*, *y*) (Figure 2). The value of the integral image at point (*x*; *y*) is the sum of all the pixels above and to the left. Consider the following:

(2) $$\begin{array}{c} ii\left(x,y\right)=\sum_{x^{\prime }\leq x,y^{\prime }\leq y}i\left(x^{\prime },y^{\prime }\right), \end{array}$$

where *ii*(*x*; *y*) is the integral image and *i*(*x*; *y*) is the original image using the following pair of recurrences:

(3) $$\begin{array}{c} s\left(x,y\right)=s\left(x,y-1\right)+i\left(x,y\right),\\ ii\left(x,y\right)=ii\left(x-1,y\right)+s\left(x,y\right). \end{array}$$

The integral image can be computed in one pass over the original image.

Figure 2 demonstrates the calculation method of summed area table. This is the reason that Adaboost calculates feature using this technique. For example by using only four array references, the sum of the pixels within rectangle D can be calculated according to the following way:

 at location 1 (sum of the pixels in rectangle A);
 at location 2 (A + B);
 at location 3 (A + C);
 at location 4 (A + B + C + D);
 finally the sum within rectangle D is 4 + 1 − (2 + 3).

#### 3.2.2. Gentle Adaboost Algorithm (GAB)

In this research we used Gentle Adaboost algorithm (GAB) [1, 12] to train a number of haar-like features (over 85000) using haar-cascade and train-cascade methodologies. Among four different types of Adaboost algorithm, in real Adaboost algorithm, logarithm of the sample's posterior probability is applied to check the competent weak classifier, which will greatly boost the weight of “noise” in the training set. But, “noise” samples are difficult to be completely eliminated, which leads to overfitting during training stage. As a result, the node classifier's generalization ability will be weakened. In order to improve the node classifier's generalization ability, Gentle Adaboost has been utilized in [1]. The pseudocode of the algorithm is as follows.

(a) Let (*x*_1_, *y*_1_) ⋯ (*x*_*n*_, *y*_*n*_) be example images where *y*_*i*_ = −1,1 for negative and positive examples accordingly.
(b) Now the weights needed to be initialized:
 
*w*_1,*i*_ = 1/2*p*, 1/2*q* for *y*_*i*_ = −1,1; accordingly *p* and *q* are the numbers of negatives and positives.
(c) For *t* = 1 ⋯ *T*, consider the following.
  (1) Weights normalization is
    (4) $$\begin{array}{c} w_{l,i}⟵\frac{w_{t,i}}{\sum_{j=1}^{n}w_{t,j}}. \end{array}$$
  (2) For each feature *j*, train a classifier *h*_*j*_ which is limited to use a single feature. The error is evaluated with respect to *w*_*i*_, *ϵ*_*j*_ = ∑_*i*_ *w*_*j*_ | *h*_*j*_(*x*_*i*_) − *y*_*i*_)|.
  (3) Classifier (*h*_*t*_) should be chosen with minimum error rate *ϵ*_*t*_.
  (4) Weights update is *w*_*t*+1,*i*_ = *w*_*t*,*i*_ *β*_*t*_^1−*e*_*i*_^.
   While *e*_*i*_ = 0 if example *x*_*i*_ is classified correctly, *e*_*i*_ = 1 otherwise and *β*_*t*_ = *ϵ*_*t*_/(1 − *ϵ*_*t*_).
  (5) The strong classifier is
    (5) $$\begin{array}{c} h\left(x\right)=\{\begin{array}{cc} 1 & \overset{T}{\underset{t=1}{\sum }}\alpha_{t}h_{t}\left(x\right)\geq \frac{1}{2}\overset{T}{\underset{t=1}{\sum }}\alpha_{t}\\ -1 & \text{Otherwise}, \end{array} \end{array}$$
   where *α*_*t*_ = log⁡(1/*β*_*t*_).

#### 3.2.3. Boosted Haar-Cascade

It is a built-in package of OpenCv [12], which supports only haar-like features [16]. The main focus of this method is the accuracy of object detection and less false detection. The word “cascade” means that the resultant classifier consists of several simpler classifiers that are applied subsequently to a region of interest until at some stage the candidate is barred or all the stages are passed. The word “boosted” means that the classifiers at every stage of the cascade are complex themselves and they are built out of basic classifiers using one of four different boosting techniques (weighted voting). Currently Discrete Adaboost, Real Adaboost, Gentle Adaboost, and Logitboost are supported. In this research Gentle Adaboost (GAB) has been applied to improve classifier's generalization ability.

#### 3.2.4. Boosted Train-Cascade

OpenCV train-cascade package supports both the haar-like features [16] and LBP (local binary pattern) [18] and the multicore platform for object detection [18]. The main focus of this method is faster detection. There is a drawback, that is, substantial false positive rate. Without this limitation this method would be more suitable for object detection. The main difference between haar-cascade and train-cascade is the structure of feature set data. Train-cascade uses binary data for storing feature set whether haar-cascade uses double type data [12, 15, 19].

## 4. Experiment

**Figure 3:**
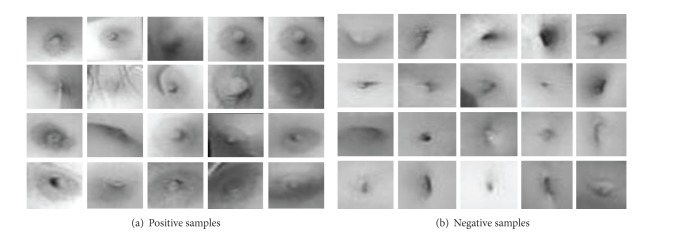
Training samples.

The OpenCV library is designed to be used in conjunction with applications that pertain to the field of human computer interaction (HCI), biometrics, robotics, image processing, and other computer vision related areas where visualization is important and includes an implementation of haar-classifier detection and training [8]. To train the classifiers, two sets of images are needed. One set contains an image or scene that contains the object of interest, in this case a nipple feature, which is going to be detected. This set of images is referred to as the positive images. The other set of images, the negative images, contains one or more instances of the object. The location of the objects within the positive images is specified by the image name, the upper left pixel, and the height and width of the object [16]. In this research we used Gentle Adaboost haar-cascade and train-cascade classifiers for training nipple dataset. We have 1198 positive training samples and 1995 negative images. At first positive images were filtered using YCbCr skin color model, after nipple objects were cropped and scaled to 20 × 20 pixels. This would help significant false minimization. For faster computation we used Gentle Adaboost (GAB) classifier. Minimum hit rate and maximum false alarm were set as 0.995 and 0.5, respectively. After training 1155 weak classifiers, we obtained 15 staged strong Gentle Adaboost classifiers.  Figure 3 shows some cropped positive and negative nipple images.

## 5. Results

**Figure 4:**
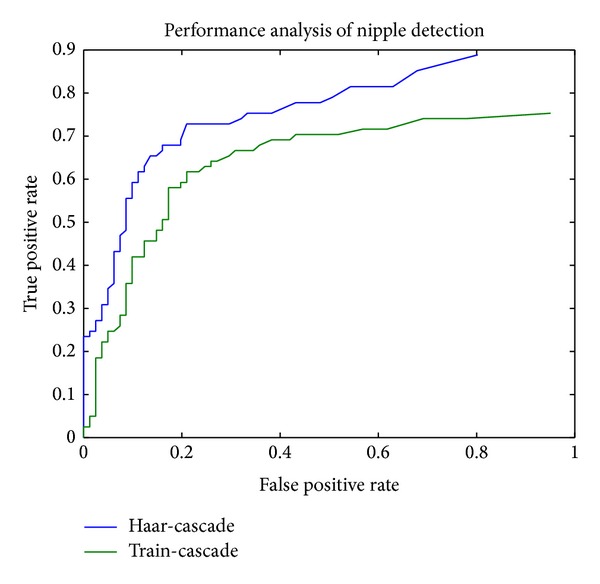
ROC for two classifiers.

Figure 4 illustrates the robustness of our experiments. The performance was illustrated through a receiver operating characteristics (ROC) curve. We tested our classifier with 400 classified nipple images and 125 nonnipple images. It is shown that the performance is better for haar-cascade classifier, but in order to satisfy detection time train-cascade performed well. For instance, Haar-cascade classifier takes 0.162 seconds for checking each positive sample, while train-cascade needs 0.127 seconds.

### 5.1. Comparison with Existing Nipple Detection Methods

According to the review there was only three papers published based on nipple detection. A comparative analysis between existing methods and our methods is shown in Table 1.

Table 1 documents a comparative analysis on detection rate, false positive rate, and false negative rate between three existing methods and our two proposed methods using Gentle Adaboost haar-cascade and train-cascade. Gentle Adaboost haar-cascade outperformed the highest detection rate and lowest false negative rate. The lowest false positive rate was achieved by using self-organizing map [7] but it has a significant false negative rate.

## 6. Conclusion

Obscenity is a vital issue for Internet safety. For ensuring safe browsing, researchers are working hard to find a concrete methodology. Unfortunately it is impossible and hence there are a large number of different techniques available to address this issue. Existing systems are mainly focused on skin color tones. The main problem of those techniques is huge false detection due to skin-like objects and color. Also it identifies nudity with partially exposed images. In this situation erotogenic human body parts detection technique solves the problems. The literature was addressed only on human body parts. In our research we combined skin color and a vital part of human body part, which can address offensive images easily. In this paper we tried to develop a novel method for accurately detecting nipples from pornographic images. Exposed nipples are considered erotogenic human body parts and vital issue for nudity. Our aim was to filter that kind of offensive images. Here, haar-cascade and train-cascade methods were analyzed using Gentle Adaboost algorithm and it was found that haar-cascade performed well in accordance with accuracy and train-cascade improves speedup of detection process. Moreover, skin filter prior to training made our system more robust and eliminated significant number of false images. Our experimental results are better than three prior works on nipple detection (Table 1), but still there is some false detection. This limitation can be overcome by using some heterogeneous classifiers with appropriate large dataset.

## Figures and Tables

**Table 1:** Strength and weakness of different nipple detection methods.

Methods	Detection rate (%)	False positive (FP) %	False negative (FN) %
Self-organizing map (SOM) [10]	65.40	0.22	34.60
Adaboost [20]	75.64	17.40	24.40
Cascaded Adaboost (haar-cascade) [21]	90.37	7.46	4.86
Gentle Adaboost with haar-cascade (our approach)	98.75	1.00	1.25
Gentle Adaboost with train-cascade (our approach)	84.29	22.22	15.71
